# A review on the role of MEG8 lncRNA in human disorders

**DOI:** 10.1186/s12935-022-02705-9

**Published:** 2022-09-16

**Authors:** Soudeh Ghafouri-Fard, Tayyebeh Khoshbakht, Bashdar Mahmud Hussen, Mohammad Taheri, Seyedpouzhia Shojaei

**Affiliations:** 1 Department of Medical Genetics, School of Medicine, Shahid Beheshti University of Medical Sciences, Tehran, Iran; 2 Phytochemistry Research Center, Shahid Beheshti University of Medical Sciences, Tehran, Iran; 3 Department of Pharmacognosy, College of Pharmacy, Hawler Medical University, Kurdistan Region, Erbil, Iraq; 4 Center of Research and Strategic Studies, Lebanese French University, Erbil, Kurdistan Region Iraq; 5 Urology and Nephrology Research Centre, Shahid Beheshti University of Medical Sciences, Tehran, Iran; 6 Department of Critical Care Medicine, Imam Hossein Medical and Educational Center, Shahid Beheshti University of Medical Sciences, Tehran, Iran

**Keywords:** MEG8, lncRNA, cancer, Expression, Biomarker

## Abstract

Maternally expressed 8 (MEG8) is a long non-coding RNA which is expressed in the nucleus. It is highly expressed in adrenal, placenta and brain. Recent studies have shown contribution of MEG8 in different disorders ranging from neoplastic ones to diabetic nephropathy, atherosclerosis, ischemic stroke, trophoblast dysfunction and abortion, Henoch-Schonlein purpura and osteoarthritis. It has an oncogenic role in the development of lung, pancreatic and liver cancer. In the current review, we summarize the role of this lncRNA in mentioned disorders, based on the evidence obtained from in vitro, in vivo and human studies.

## Introduction

Long non-coding RNAs (lncRNAs) have been largely investigated for their contribution in human disorders, particularly cancer [1]. These transcripts have sizes more than 200 nucleotides, do not possess considerable open reading frames and regulate gene expression through diverse epigenetic mechanisms. They participate in transcriptional and post-transcriptional regulation via interacting with DNA, RNA or proteins [2]. Moreover, they are involved in the regulation of mRNA splicing and can serve as precursors for microRNAs (miRNAs) [3]. Thus, lncRNAs regulate gene expression at almost all levels.

Maternally expressed 8 (MEG8), alternatively named as RNA Imprinted and Accumulated in Nucleus (Rian), is an example of which is expressed in the nucleus [4, 5]. In human, *MEG8* gene resides in a cluster of imprinted genes on chromosome 14q32.3. The encoded transcript is has a preferential expression from the maternal allele in skeletal muscle, and seems to be regulated in a coordinate manner with other imprinted genes in this genomic area (https://www.ncbi.nlm.nih.gov/gene/79104). It is highly expressed in adrenal, placenta and brain [6]. This small nucleolar RNA host gene has 52 known splice variants (https://asia.ensembl.org/Homo_sapiens/Gene/Splice?db=core;g=ENSG00000225746;r=14:100894770-101038859).

Recent studies have shown contribution of MEG8 in different disorders ranging from neoplastic ones to diabetic nephropathy, atherosclerosis, ischemic stroke, trophoblast dysfunction and abortion, Henoch-Schonlein purpura and osteoarthritis. In the current review, we summarize the role of this lncRNA in mentioned disorders, based on the evidence obtained from in vitro, in vivo and human studies.

### ***In vitro*studies**

Liu et al. have investigated function of MEG8 in lung cancer. For this purpose, they have transfected lung epithelial BEAS-2B cells with MEG8 overexpressing vector. Moreover, they have transfected lung cancer A549 and H1299 cells with MEG8 or miR-107 overexpressing vectors as well as knockdown plasmids. Up-regulation of MEG8 has increased proliferation, migration and invasion of lung epithelial cells. On the other hand, MEG8 knockdown or miR-107 up-regulation has blocked cell progression of lung cancer cells. Their functional studies have confirmed competitive binding of MEG8 and CDK6 with miR-107 and their function in regulation of progression of lung cancer. In addition, MEG8 knockdown or miR-107 overexpression could suppress Rb and E2F3 phosphorylation. Taken together, MEG8 could enhance progression of lung cancer through regulation of miR-107/CDK6 axis and activation of Rb/E2F3 pathway [7]. Another study in lung cancer has shown up-regulation of MEG8, parallel with down-regulation of miR-15a-5p and miR-15b-5p in cancer cell lines. MEG8 silencing has suppressed proliferation, migration, and invasion of lung cancer cells through targeting miR-15a-5p/miR-15b-5p [8].

Terashima et al. have shown induction of expression of MEG8 in the course of TGF-β-mediated epithelial-mesenchymal transition (EMT) in both lung and pancreatic cancer cells. Up-regulation of MEG8 could suppress expression of miR-34a and miR-203, leading to over-expression of SNAI1 and SNAI2 transcription factors and subsequent repression of cadherin 1/E-cadherin. Mechanistically, MEG8 interacts with EZH2 protein and increases recruitment of EZH2 to the regulatory sections of miR-34a and miR-203. EZH2 enhances histone H3 methylation in these regions and suppress transcription of these miRNA genes. Concurrent expression of MEG8 and MEG3 can increase EMT-associated alterations in cell morphology and increase motility of cells in the absence of TGF-β. Taken together, MEG8 participates in the induction of EMT through epigenetic mechanisms [9].

**Fig. 1:**
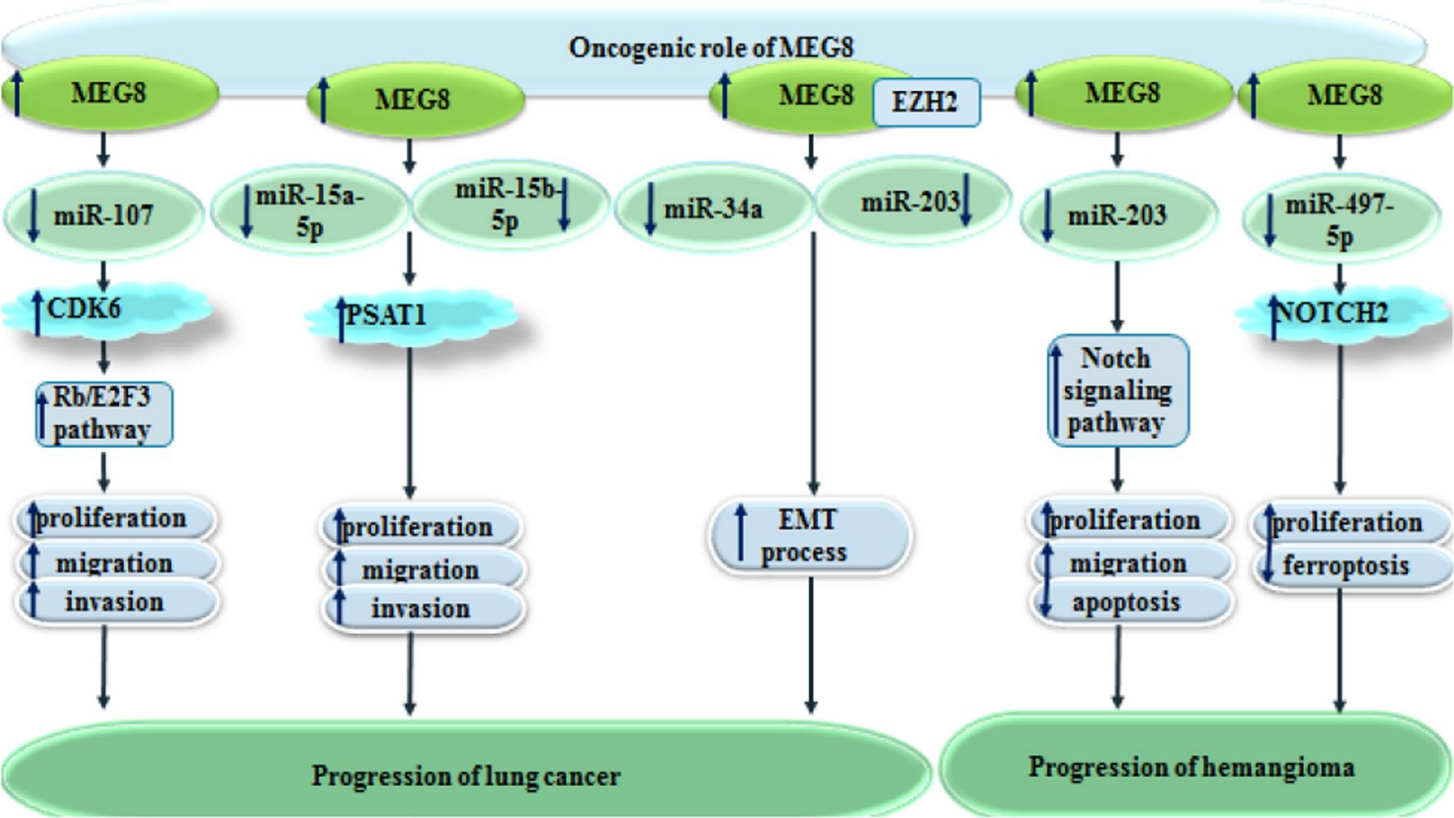
Oncogenic roles of MEG8 in lung cancer and hemangioma

The effects of MEG8 silencing have also been investigated in human hemangioma endothelial cells. Notably, MEG8 silencing has suppressed proliferation of these cells and increased their apoptosis through modulation of the effects of miR-203 on JAG1 and Notch expressions [10]. Figure 1 summarizes the effect of MEG8 in the pathogenesis of lung cancer and hamangioma.

Expression of MEG8 has been found to be elevated in hepatocellular carcinoma (HCC) cells. MEG8 silencing has significantly suppressed the proliferative and invasive abilities of these cells. Furthermore, MEG8 has been shown to sponge miR-367-3p to increase 14-3-3ζ levels, suppress degradation of TGFβR1, and promote TGF-β signaling [11].

**Fig. 2:**
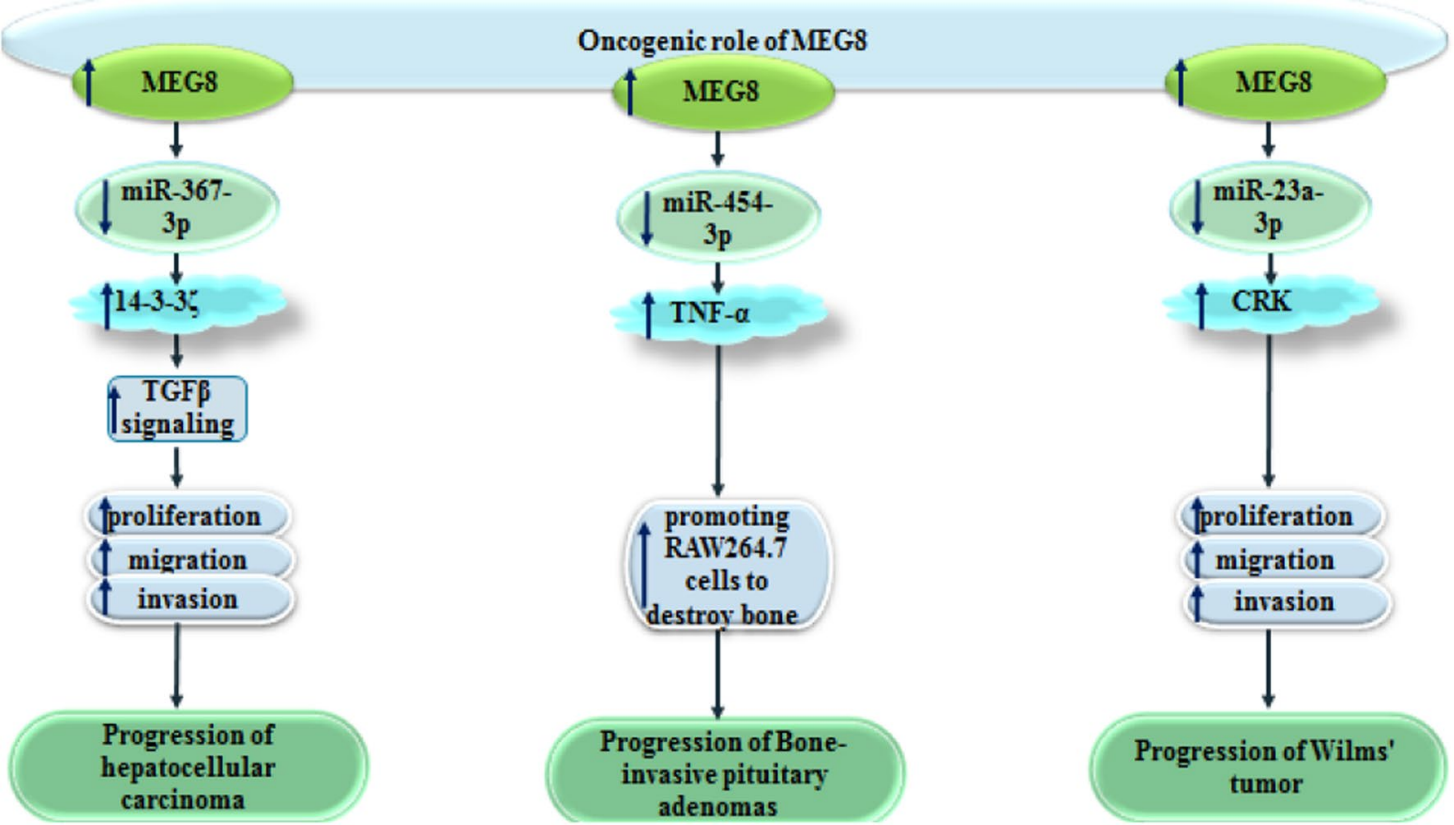
Oncogenic roles of MEG8 in hepatocellular carcinoma, bone invasive pituitary adenoma and Wilms tumor

Moreover, MEG8 has been shown to participate in the progression of bone-invasive pituitary adenoma through sponging miR-454-3p and increasing TNF-α expression [12]. Similarly, expression of MEG8 has been found to be elevated in Wilms tumor cells, parallel with up-regulation of CRK and down-regulation of miR-23a-3p. MEG8 silencing or miR-23a-3p up-regulation has blocked viability, migration potential and invasive properties of these cells. Mechanistically, MEG8 binds with miR-23a-3p to release CRK from inhibitory effects of this miRNA. Taken together, MEG8 regulates pathogenesis of Wilms tumor through miR-23a-3p/CRK axis [13]. Figure 2 shows the oncogenic roles of MEG8 in hepatocellular carcinoma, bone invasive pituitary adenoma and Wilms tumor.

Exposure of podocyte cells with high-glucose conditions has led to over-expression of MEG8 and miR-770-5p. In fact, up-regulation of MEG8 increases miR-770-5p levels through decreasing methylation of the miR-770-5p gene. Up-regulation of MEG8 and miR-770-5p can increase cell apoptosis under high-glucose conditions. Taken together, MEG8 can increase miR-770-5p levels via epigenetic mechanism to induce diabetic nephropathy through enhancing cell apoptosis [14].

MEG8 can also contribute in the pathogenesis of other non-neoplastic conditions. For instance, it participate in the pathoetiology of atherosclerosis through regulation of proliferation, migration and apoptosis of vascular smooth muscle cells via affecting expression of PPARα [15]. This lncRNA can attenuate cerebral ischemia following ischemic stroke via influencing miR-130a-5p/VEGFA axis [16].

Over-expression of MEG8 in trophoblast cells has reduced proliferation and invasion of these cells, while its silencing has exerted the opposite effects. This imprinted lncRNA participates in the modulation of function of trophoblast cells during early stages [17].

MEG8 can also contribute in the pathogenesis of Henoch Schonlein purpura through sponging miR-181a-5p, influencing levels of SHP2 expression and increasing M1 macrophage polarization [18]. Finally, this lncRNA can regulate proliferation and apoptosis of chondrocytes, thus participating in the pathogenesis of osteoarthritis [19].

MEG8 has also been shown to contribute to the pathoetiology of cardiovascular diseases through epigenetic mechanisms. In vitro studies have demonstrated that MEG8 knock-down impairs angiogenic sprouting and reduces proliferation of HUVEC cells. RNA sequencing experiments have shown up-regulation of the inhibitor of angiogenesis TFPI2 after MEG8 silencing. From a mechanistical point of view, MEG8 silencing can lead to a decrease in H3K27me3 marks at the TFPI2 promoter [20]. Table 1 shows summary of in vitro studies about the role of MEG8 in human disorders.

**Table 1:** Summary of in vitro studies about the role of MEG8 in human disorders (∆: knock-down or deletion, VSMC: vascular smooth muscle cell, OGD: oxygen-glucose deprivation)

Tumor/ disorder type	Targets/ Regulators and Signaling Pathways	Cell line	Function	References
Lung cancer	miR-107, CDK6, Rb/E2F3 pathway	BEAS-2B, A549 and H1299	↑ MEG8: ↑ proliferation, ↑ migration, ↑ invasion ∆ MEG8: ↓ cell progression	[7]
	miR-15a-5p, miR-15b-5p, PSAT1	16HBE, A549, H1299, H1975, SPC-A1, and PC-9	∆ MEG8: ↓ proliferation, ↓ migration, ↓ invasion	[8]
	miR-34a and miR-203, EZH2, SNAI1 and SNAI2	A549 and LC2/ad	MEG8 was involved in EZH2 recruitment to inhibit miR-34a and miR-203 expression. ↑ MEG8: ↑ EMT process	[9]
Pancreatic cancer	miR-34a and miR-203, EZH2, SNAI1 and SNAI2	Panc1	MEG8 was involved in EZH2 recruitment to inhibit miR-34a and miR-203 expression. ↑ MEG8: ↑ EMT process	
Hemangioma	miR203, Notch signaling pathway	HemECs	∆ MEG8: ↓ proliferation, ↓ migration, ↑ apoptosis	[10]
	miR-497-5p, NOTCH2	HemECs	∆ MEG8: ↓ proliferation, ↑ ferroptosis	[21]
Hepatocellular carcinoma	miR-367-3p, 14-3-3ζ, TGFβR1, TGFβ signaling	Human LO2 hepatocytes and HepG2, Huh7, HCCLM3, and HMCC-97 H HCC	∆ MEG8: ↓ proliferation, ↓ migration, ↓ invasion	[11]
Bone-invasive pituitary adenomas	miR-454-3p, TNF-α	293T and RAW264.7	↑ MEG8: ↑ promoting RAW264.7 cells to destroy bone	[12]
Wilms’ tumor	miR-23a-3p, CRK	WT cells	∆ MEG8: ↓ viability, ↓ migration, ↓ invasion	[13]
Diabetic nephropathy	miR-770-5p	CIHP-1	↑ MEG8: ↑ glucose-mediated apoptosis, ↑ miR-770-5p expression by reducing the methylation of miR-770-5p	[14]
Atherosclerosis	miR-181a, PPARα	VSMCs	↑ MEG8: ↓ VSMC proliferation, ↓ migration, ↑ apoptosis	[15]
Ischemic stroke	miR-130a-5p, VEGFA	OGD-treated BMECs	∆ MEG8: ↓ viability, ↓ migration, ↓ angiogenesis	[16]
Trophoblast dysfunction and abortion		HTR-8/SVneo cell line from early villous trophoblasts (EVTs)	↑ MEG8: ↓ proliferation of trophoblast, ↓ invasion	[17]
Henoch-Schonlein purpura	miR-181a-5p, SHP2,	RMDMs from HSP rats	↑ MEG8: ↑ M1 polarization, ↓ JAK2/STAT3 pathway	[18]
Osteoarthritis	PI3K/AKT signaling pathway	IL-1β-treated C28/I2 cells	∆ MEG8: ↓ proliferation, ↓ activation of the PI3K/AKT signaling pathway, ↑ apoptosis, ↑ inflammatory response	[19]

## Animal studies

Studies in xenograft models of lung cancer have confirmed that MEG8 enhances tumor growth through modulation of miR-15a/b-5p/PSAT1. MEG8 silencing has considerably decreased tumor growth and burden in animal models of lung cancer [8]. This lncRNA has a similar effect bone-invasive pituitary adenomas [12].

Contribution of MEG8 in the pathogenesis of trophoblast dysfunction and abortion has been investigated in an animal study. Sheng et al. have obtained placental samples from pregnant female mice at three important developmental stages and assessed lncRNA signature using microarray technique. They have shown that Meg8 might have a crucial role in this process [17]. Table 2 shows summary of in vivo studies about the role of MEG8 in diverse disorders.

**Table 2:** Summary of in vivo studies about the role of MEG8 in diverse disorders (∆: knock-down or deletion)

Tumor/ disorder Type	Animal models	Results	References
Lung cancer	Nude mice	∆ MEG8: ↓ tumor growth, tumor volume, tumor weight	[8]
Bone-invasive pituitary adenomas	Male BALB/c nude mice	↑ MEG8: ↑ tumor volume, ↑ bone destruction	[12]
Ischemic stroke	Male Sprague–Dawley rats	↑ MEG8: ↓ cerebral ischemia in ischemic stroke	[16]
Trophoblast dysfunction and abortion	Pregnant C57BL/6 female mice	MEG8 expression gradually increased during placental development.	[17]
Henoch-Schonlein purpura	Wistar rats	Expression levels of MEG8 were much lower in HSP rats than control rats.	[18]

## Studies in clinical samples

A comprehensive assessment of transcriptome profile and clinical features has led to identification of a ceRNA network that has prognostic impact in uterine corpus endometrial carcinoma. MEG8 has been among 10 lncRNAs that have been related with prognosis of this type of cancer [22].

Expression of MEG8 has been found to be increased in tissue samples obtained from lung cancer patients compared to corresponding normal tissues. Notably, expression of this lncRNA has been negatively correlated with expression levels of miR-15a-5p and miR-15b-5p in these cancerous samples [8]. In HCC patients, over-expression of MEG8 has been correlated with the poor prognosis, edmondson Steiner grading, venous infiltration, and the number of tumor nodules [11]. In Wilms tumor samples, expression of MEG8 has been correlated with histological subtype, lymphatic invasion, and National Wilms Tumor Study (NWTS) stage [13]. On the other hand, an in silico approach in ovarian cancer has shown that the expression of MEG8 is associated with to better overall survival in Kaplan-Meier analysis. This approach has led to identification of the MEG8/miR-378d/SOBP axis as a functional axis in progression and prognosis of ovarian cancer [23]. Similar to ovarian cancer, expression of MEG8 has been found to be down-regulated in colorectal cancer samples compared with controls. This trend has also been demonstrated in the precancerous colonic lesions [24]. Moreover, Meg8 has been shown to have lower expression in neoplastic stromal cell population of giant cell tumors compared with mesenchymal stem cells [25].

**Fig. 3:**
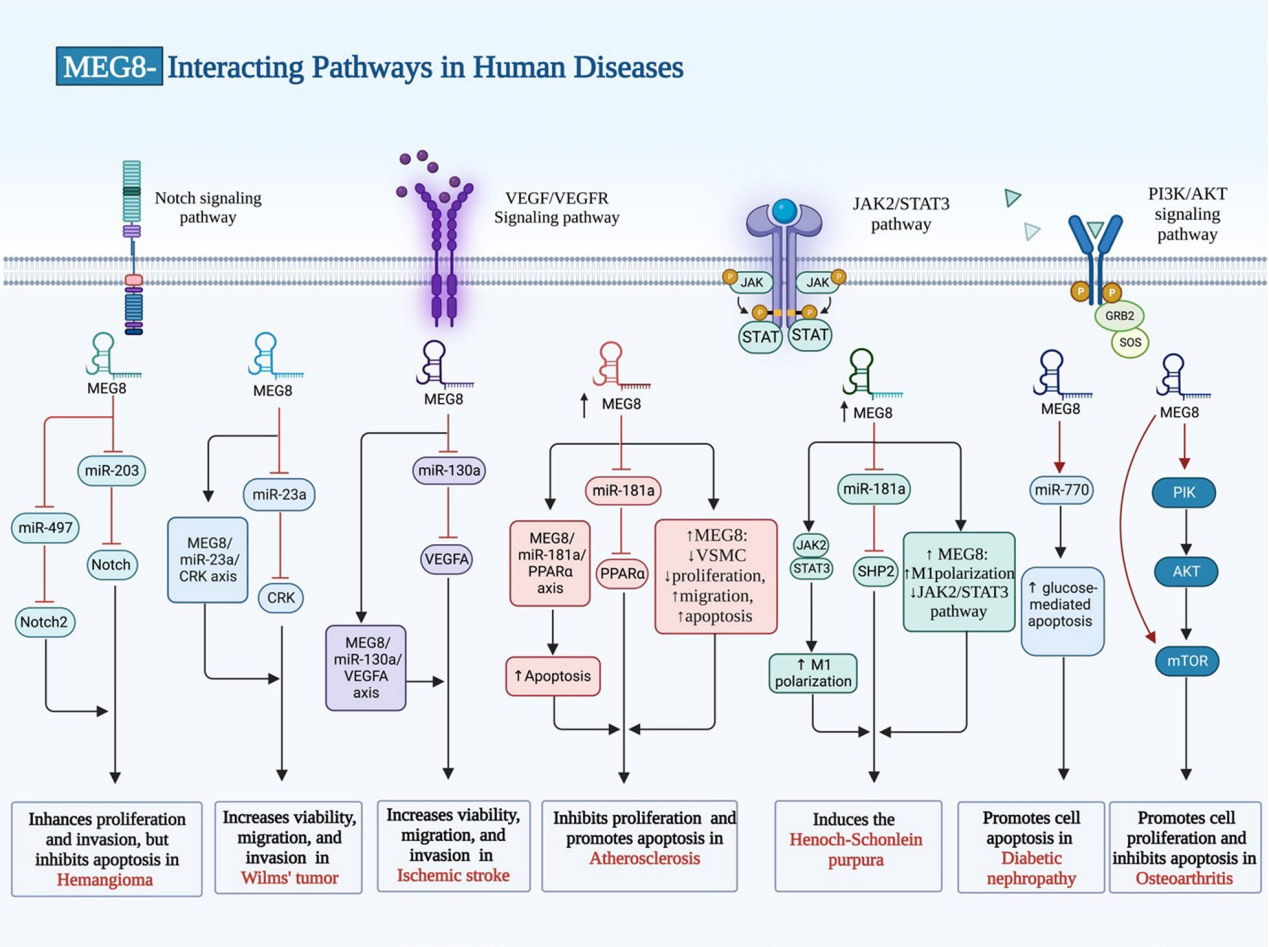
Summarizes the effect of MEG8 lncRNA in the pathogenesis of different types of human diseases

Expression of MEG8 and miR-770-5p has been shown to be increased in plasma of diabetic patients, particularly in those with diabetic [14] (Fig. 3). Moreover, this lncRNA has a possible role in trophoblast dysfunction, since it is over-expressed in human spontaneous abortion villi. Moreover, methylation of its promoter region has been shown to be elevated in spontaneous abortion villus samples [17]. Table 3 shows the results of studies that reported dysregulation of MEG8 in clinical samples.

**Table 3:** Results of studies that reported dysregulation of MEG8 in clinical samples

Tumor/ disorder type	Samples	Expression (Tumor vs. Normal)	Kaplan-Meier analysis (impact of MEG8 up-regulation)	Univariate/ Multivariate cox regression	Association of MEG8 expression with Clinicopathologic characteristics	References
Lung cancer	21 pairs of NSCLC tissues and ANCTs	Up	–	–	–	[7]
	37 pairs of NSCLC tissues and ANCTs	Up	–	–	–	[8]
Hepatocellular carcinoma	74 pairs of NSCLC tissues and ANCTs	Up	Lower OS and DFS	–	Edmondson Steiner grading, venous infiltration, and the number of tumor nodules	[11]
Bone-invasive pituitary adenomas	40 pituitary adenoma patients	Up	–	–	–	[12]
Ovarian cancer	GEO database: (GSE36668, GSE12470, GSE14407, and GSE27651), GEPIA2 and starBase database	Down	Better OS	–	–	[23]
Colorectal cancer	20 colorectal cancer, 20 adenomas, 20 healthy controls	Lower in colorectal cancer and adenomas than controls	–	–	–	[24]
Giant cell tumor of bone	5 GCTSCs and 5 MSCs	Down in GCTSCs	–	–	–	[25]
Diabetic nephropathy	66 DN patients 66 DM patients 66 healthy controls	Up in DN and DM than controls, Up in DN than DM	–	–	–	[14]
Gestational diabetes mellitus	400 pregnant females (78 females were diagnosed as GDM during pregnancy)	Up in females who showed GDM	–	–	One month before the diagnosis of GDM, plasma levels of MEG8 were sufficient to distinguish GDM patients from healthy controls. GDM females with higher level of MEG8 showed higher incidence of kidney injury.	[26]
Trophoblast dysfunction and abortion	20 spontaneous abortion villi in early pregnancy and 20 normal early villi	Up in spontaneous abortion villi	–	–	–	[17]
Temple syndrome	3 Temple syndrome patients	DNA-hypermethylation of the MEG8-DMR was observed in 3 patients.	–	–	–	[27]
	13 non-deletion TS14 patients	MEG8-DMR was hypermethylated in all patients.	–	–	–	[28]
Kagami-Ogata syndrome (KOS14)	4 KOS14 patients with different deletions	MEG8-DMR was hypomethylated in patients.	–	–	–	
Osteoarthritis	22 OA patients and 22 healthy controls	Down	–	–	–	[19]
Abnormal semen	40 Semen samples from patients (8 normozoospermic, 16 asthenospermic, 3 oligospermic, 11 oligoasthenospermic and 2 morphologically deformed)	DMR of MEG8 were different in the abnormal semen groups. MEG8 DMR methylation was significantly increased in the asthenospermic group. Higher methylation levels of MEG8 DMR in the oligospermic and oligoasthenospermic groups were observed.	–	–	–	[29]

*ANCTs* adjacent non-cancerous tissues,* NSCLC* non-small cell lung cancer,* OS* overall survival,* DFS* disease-free survival,* GCTSCs* neoplastic stromal,* MSC* mesenchymal stem cell,* DN* diabetic nephropathy,* DM* diabetes mellitus,* GDM* gestational diabetes mellitus,* DMR* differentially methylated region,* TS14* Temple syndrome,* OA* Osteoarthritis

## Discussion

MEG8 participates in the pathoetiology of different disorders ranging from neoplastic ones to diabetic nephropathy, atherosclerosis, ischemic stroke, trophoblast dysfunction and abortion, Henoch-Schonlein purpura and osteoarthritis. It has an oncogenic role in the development of lung, pancreatic and liver cancer. However, in ovarian and colorectal cancers its expression has opposite trend.

Functional effects of MEG8 up-regulation/silencing have investigated in different contexts. This lncRNA has interactions with a variety of miRNAs such as miR-107, miR-15a-5p, miR-15b-5p, miR-34a and miR-203, miR-497-5p, miR-367-3p, miR-454-3p, miR-23a-3p, miR-770-5p, miR-181a, miR-130a-5p and miR-181a-5p. In addition to serving as molecular sponge for a number of miRNAs, MEG8 can enhance recruitment of EZH2 to the regulatory regions of miRNAs, thus regulating their expression. In fact, the regulatory role of MEG8 on expression of miR-34a and miR-203 is exerted through recruitment of EZH2 [9]. Moreover, MEG8 has been shown to increase miR-770-5p levels through decreasing methylation of the miR-770-5p gene [14]. Thus, the regulatory effects of MEG8 on miRNAs can be exerted through different mechanisms.

Although dysregulation of MEG8 has been reported in different neoplastic and non-neoplastic conditions, the diagnostic value of this lncRNA has not been investigated. Moreover, since it has different expression trends in different malignancies, future studies are needed to find the underlying mechanism of its contribution in different tumors and assess the presence of tissue-specific mechanisms.

## Conclusion

A single study in patients with Temple syndrome has shown that DNA-methylation of the MEG3- and MEG8- differentially methylated region depends on the DNA-methylation pattern of the IG-differentially methylated region [27]. However, the functional link between these differentially methylated regions has not been investigated in other contexts. Thus, future studies are needed to find the mechanism of dysregulation of MEG8 in different pathological contexts.

## Data Availability

The analyzed data sets generated during the study are available from the corresponding author on reasonable request.
